# Development of a Bacteriophage Cocktail to Constrain the Emergence of Phage-Resistant *Pseudomonas aeruginosa*

**DOI:** 10.3389/fmicb.2020.00327

**Published:** 2020-03-04

**Authors:** Yuhui Yang, Wei Shen, Qiu Zhong, Qian Chen, Xuesong He, Jonathon L. Baker, Kun Xiong, Xiaoling Jin, Jing Wang, Fuquan Hu, Shuai Le

**Affiliations:** ^1^Department of Microbiology, Army Medical University, Chongqing, China; ^2^Department of Medical Laboratory, The General Hospital of Western Theater Command, Chengdu, China; ^3^Department of Clinical Laboratory, Daping Hospital, Army Medical University, Chongqing, China; ^4^Biomedical Analysis Center, Army Medical University, Chongqing, China; ^5^The Forsyth Institute, Cambridge, MA, United States; ^6^Genomic Medicine Group, J. Craig Venter Institute, La Jolla, CA, United States

**Keywords:** dsRNA bacteriophage, phage cocktail, *Pseudomonas aeruginosa*, phage resistance, antibiotic resis

## Abstract

With the emergence of multidrug-resistant and extensively drug-resistant bacterial pathogens, phage therapy and other alternative or additional therapeutic modalities are receiving resurgent attention. One of the major obstacles in developing effective phage therapies is the evolution of phage resistance in the bacterial host. When *Pseudomonas aeruginosa* was infected with a phage that uses O-antigen as receptor, phage resistances typically achieved through changing or loss of O-antigen structure. In this study, we showed that dsRNA phage phiYY uses core lipopolysaccharide as receptor and therefore efficiently kills the O-antigen deletion mutants. Furthermore, by phage training, we obtained PaoP5-m1, a derivative of dsDNA phage PaoP5, which is able to infect mutants with truncated O-antigen. We then generated a cocktail by mixing phiYY and PaoP5-m1 with additional three wide host range *P. aeruginosa* phages. The phage cocktail was effective against a diverse selection of clinical isolates of *P. aeruginosa*, and in the short-term constrained the appearance of the phage-resistant mutants that had beleaguered the effectiveness of single phage. Resistance to the 5-phage cocktail emerges after several days, and requires mutations in both *wzy* and *migA* Thus, this study provides an alternative strategy for designing phage cocktail and phage therapy.

## Introduction

*Pseudomonas aeruginosa* is a common opportunistic pathogen that causes infections of the bloodstream, urinary tract, burn wounds, and is one of the major pathogens infecting the airways of cystic fibrosis patients and *P. aeruginosa* infections can be life-threatening (Jonckheere et al., 2018; Waters and Grimwood, 2018). Moreover, *P. aeruginosa* strains are frequently resistant to multiple classes of antibiotics (Lopez-Causape et al., 2018), and *P. aeruginosa* is a member of the ESKAPE pathogens (Boucher et al., 2009), which include six pathogens with well-recognized abilities to develop antibiotic resistance and cause deadly clinical outbreaks. With the emergence of multidrug-resistant isolates of *P. aeruginosa* (Sun et al., 2013; Lopez-Causape et al., 2018), phage therapy has received renewed attention (Forde and Hill, 2018; Jault et al., 2018; Kortright et al., 2019), and is a promising alternative approach for treating recalcitrant *P. aeruginosa* infections (Roach et al., 2017; Waters et al., 2017; Forti et al., 2018; Jault et al., 2018).

An optimal phage therapeutic agent necessitates some features: low immunogenicity, strictly lytic lifestyle, no toxins or antibiotic resistant genes, a wide host range against multiple isolates of the target pathogen, and the ability to constrain the emergence of phage-resistant mutants (Pires et al., 2017; Rohde et al., 2018; Kortright et al., 2019). The host range of a phage treatment can be expanded by incorporating several phages with different host-range specificities within a cocktail. The host range and killing efficiency of phage cocktails targeting *P. aeruginosa* have been studied *in vitro* and *in vivo* (Roach et al., 2017; Forti et al., 2018). Phage resistance is a somewhat more difficult problem to address, as bacteria have evolved a number of different mechanisms to defend themselves against bacteriophages, including prevention of phage adsorption and DNA injection, restriction enzymes, and CRISPR/Cas systems (Hyman and Abedon, 2010). These mechanisms are highly effective and emerge rapidly. For example, a 6-phage cocktail formulated against a broad host range of *P. aeruginosa* initially killed with great efficiency. As expected, phage-resistant mutants grew to a high density *in vitro* after only overnight incubation (Forti et al., 2018). However, in the mice and larvae infection model, the phage cocktail resistant mutants are not observed (Forti et al., 2018). When using animal models to evaluate the efficacy of phage therapy, phage resistance is rarely observed, because the resistant mutants usually have a fitness trade-off, or the minor mutants might be cleared by the immune system. On the contrary, phage resistant is quite common *in vitro* (Kortright et al., 2019). However, in clinics, a growing number of patients infected with multidrug resistant bacteria are immunocompromized, such as AIDS patients, transplant recipients with a suppressed immune system, diabetic patients, et al. These patients might not be able to clear the minor phage resistant bacteria and result in the treatment failure. Thus, the emergence of phage resistance has been reaffirmed by experts in the field as a key issue regarding the feasibility of phage therapy (Rohde et al., 2018; Kortright et al., 2019).

**FIGURE 1:**
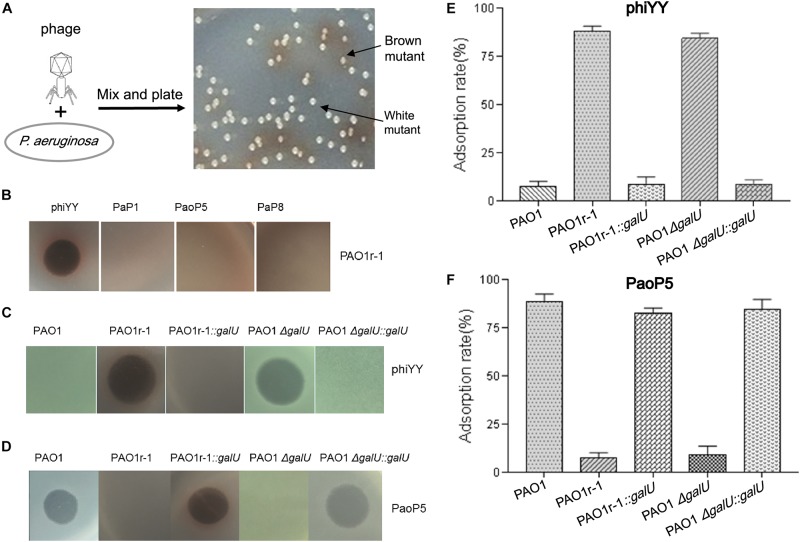
dsRNA phage phiYY infects PAO1r-1 via exposed LPS core oligosaccharides **(A)**: Colony phenotype of *P. aeruginosa* phage-resistant mutants, PAO1r-1 (brown) and PAO1w-1 (white). **(B)**: Phage spot assay indicating that dsRNA phage phiYY, but not dsDNA phages PaP1, PaoP5, and PaP8, infects PAO1r-1. Phage spot assays **(C,D)** and phage adsorption assays **(E,F)** illustrating that phiYY infects and adsorbs to PAO1-derived strains without O-antigen, including PAO1r-1 and PAO1Δ*galU*, while dsDNA phage PaoP5 infects and adsorbs to PAO1-derived strains with full O-antigen, such as PAO1 and the *galU* complement strains. (*P* < 0.05, one-way ANOVA, *n* = 3).

*Pseudomonas aeruginosa* develops phage resistance through several mechanisms, including modification or loss of the O-antigen component of lipopolysaccharide (LPS), or glycosylation of its type IV pilus (Harvey et al., 2018; Shen et al., 2018). Previously, we identified two types of phage-resistant *P. aeruginosa* mutants following infection with the dsDNA phages PaP1 or PaoP5, based upon colony pigmentation (Figure 1A). The mutant designated PAO1r-1, with a brown colony phenotype, contained a large chromosomal deletion including the genes *galU* and *hmgA*, which respectively, resulted in the complete loss of O-antigen and the accumulation of characteristic brown-colored homogentisic acids, while conferring phage resistance (Le et al., 2014; Shen et al., 2018). Meanwhile, the mutant designated PA1RG, with a white colony phenotype, contained mutations in *wzy*, an LPS biosynthesis gene, which resulted in a truncated O-antigen structure and also conferred phage resistance (Li et al., 2018). Thus, it seems mutations resulting in changes to LPS structure are likely to be a major source of phage resistance in *P. aeruginosa*. These phage-resistant mutants arose at a combined high frequency of ∼10^–5^ *in vitro* (Shen et al., 2018) and should be considered when designing a phage cocktail for eventual use in the clinical setting.

In this study, a phage cocktail designed to constrain the emergence of these phage-resistant phenotypes in *P. aeruginosa* was developed. The phage cocktail was successful in that it had a broader host range against a panel of *P. aeruginosa* clinical isolates compared to any of the tested single phages, and constrained the emergence of the previously described phage resistance phenotypes.

## Results

### The dsRNA Phage phiYY Infects Phage-Resistant PAO1r-1 With Exposed Core LPS

Previously, in *P. aeruginosa* PAO1, we identified two types of mutants that resist infection by the dsDNA phage, PaoP5 (Figure 1A). One of these, PAO1r-1 has lost the O-antigen and can be easily identified via a brown colony phenotype caused by the accumulation of homogentisic acids (Shen et al., 2018). To attempt to identify a phage that bypasses this resistance mechanism, and was able to kill PAO1r-1, a panel of lytic phages was examined (Table 1). While PAO1r-1 was resistant to dsDNA phages, including PaP1 (Lu et al., 2013), PaoP5 (Shen et al., 2016), and PaP8 (Table 1), the dsRNA phage, phiYY, was able to lyse PAOr-1 and form clear plaques (Figure 1B).

**TABLE 1:** Bacterial strains and phages used in this study.

**Strain or phage**	**Description**	**Sources**
PAO1	Wild type *P. aeruginosa* strain	Jacobs et al., 2003
PA1	Wild type *P. aeruginosa* strain	Lu et al., 2015
PAO1r-1	Phage-resistant mutant with brown pigment	Shen et al., 2018
PAO1r-1*::galU*	PAO1r-1 complemented with *galU*	This study
PAO1Δ*galU*	PAO1Δ*galU* from the transposon library	Jacobs et al., 2003
PAO1Δ*galU::galU*	PAO1Δ*galU* complemented with *galU*	This study
PAO1w-1	Phage-resistant mutant with a C1075T mutation in *wzy*	This study
PAO1w-1*::wzy*	PAO1w-1 complemented with *wzy*	This study
PAO1Δ*wzy*	Knock out Δ*wzy* in PAO1 background	This study
PAO1Δ*wzy::wzy*	PAO1Δ*wzy* complemented with *wzy*	This study
PAO1Δ*migA*	PAO1Δ*migA* from the transposon library	Jacobs et al., 2003
PAO1Δ*migA*Δ*wzy*	Knock out *wzy* in PAO1Δ*migA*	This study
phiYY	dsRNA bacteriophage isolated from Southwest hospital sewage	Yang et al., 2016
PaP1	dsDNA bacteriophage isolated from Southwest hospital sewage	Le et al., 2013
PaP8	dsDNA bacteriophage isolated from Southwest hospital sewage	This study
PaoP5	dsDNA bacteriophage isolated from Southwest hospital sewage	Shen et al., 2016
PaoP5-m1	PaoP5 mutant phage with a A715C mutation in *orf75*	This study

**FIGURE 2:**
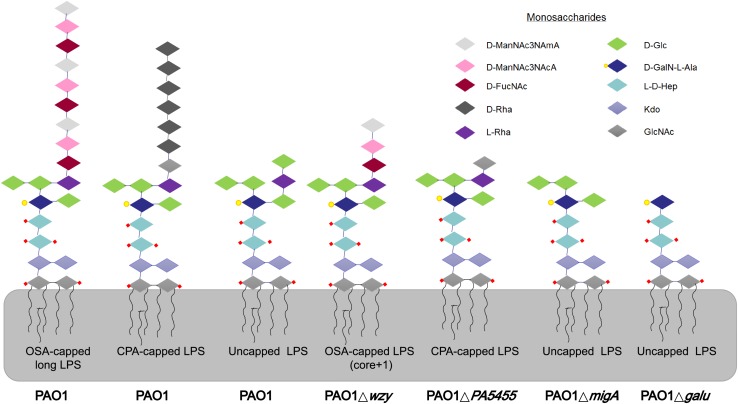
LPS glycoforms on the surface of *P. aeruginosa* strains. Wild-type PAO1 forms OSA-capped LPS, CPA-capped, and uncapped LPS. PAO1Δ*wzy* has only one copy of the typically repeating O-antigen sugar unit. PAO1Δ*galU* has no O-antigen, exposing the uncapped core. PAO1Δ*PA5455* has only GlcNAc, instead of the full-length CPA. PAO1Δ*migA* lacks the L-Rha and D-Glc normally observed on the uncapped core. d-ManNAc3NAcA:di-N-acetylated mannuronic acid; D-FucNAc: d-2-amino-2,6-dideoxy-galactose-acetamido; D-Rha: D-rhamnose; L-Rha: L-rhamnose; D-Glc: D-glucose; D-GalN-L-Ala: d-galactosamine-l-alanyl; L-D-Hep: L-glycero-d-manno-heptose; Kdo: 3-deoxy-d-manno-oct-2-ulosonic acid; GlcNAc: glucose-acetamido.

As stated above, PAO1r-1 carries a deletion in *galU* and therefore lacks the O-antigen component of LPS, leaving the core oligosaccharides of LPS exposed on the cell surface (Choudhury et al., 2005; Le et al., 2014). Accordingly, phiYY is likely to utilize the LPS core oligosaccharide instead of O-antigen as the receptor to initiate infection (Figure 2). Because PAO1r-1 contains a large chromosomal deletion, a mutant containing a single-gene disruption of *galU* (PAO1Δ*galU*), as well as complement strains restoring *galU* in both genetic backgrounds (PAO1r-1::*galU* and PAO1Δ*galU*::*galU*), were examined for susceptibility to phiYY and PaoP5. As shown in Figure 1C, phiYY lysed PAO1r-1 and PAO1Δ*galU*, but did not kill the *galU* complemented strains, indicating that loss of *galU* allows for phiYY infection. On the contrary, PaoP5 infected PAO1, PAO1r-1::*galU* and PAO1Δ*galU*::*galU*, but not the PAO1Δ*galU* mutant (Figure 1D), indicating that loss of *galU* prevents infection by PaoP5.

A phage adsorption assay was used to further investigate the binding of these phages to the bacterial strains. phiYY adsorbs to PAO1r-1 and PAO1Δ*galU* with high efficiency, but cannot bind to PAO1 or the *galU* complement strains (Figure 1E). Meanwhile, phage PaoP5 efficiently adsorbs to PAO1 and the *galU* complement strains, but not PAO1r-1 or PAO1Δ*galU* (Figure 1F). Together, these data strongly indicate that the receptor for phiYY is the core oligosaccharide, while the receptor for PaoP5 is O-antigen. Therefore, phiYY may infect *P. aeruginosa* mutants lacking an O-antigen that arise following infection by O-antigen-binding phages, such as PaoP5.

### Training the dsDNA Phage PaoP5 to Infect White Mutants With Truncated LPS

We previously identified PA1RG, a mutant of *P. aeruginosa* PA1, resistant to the dsDNA phage, PaP1, with a white colony phenotype (Li et al., 2018). This strain carries a C595T mutation in *wzy*, the B-band O-antigen polymerase, which results in a truncated O-antigen with only one copy of the normally-repeating sugar unit (Figure 2).

In this study, we isolated 5-phage-resistant PAO1 mutants with a white colony phenotype. Three out of these five isolates had mutations in *wzy* as detected by Sanger sequencing of the PCR fragment amplified from the isolates. Two mutants had *a* wzy C736G mutation and the third, designated PAO1w-1, contained a *wzy* C1075T mutation, which resulted in a premature stop codon.

**FIGURE 3:**
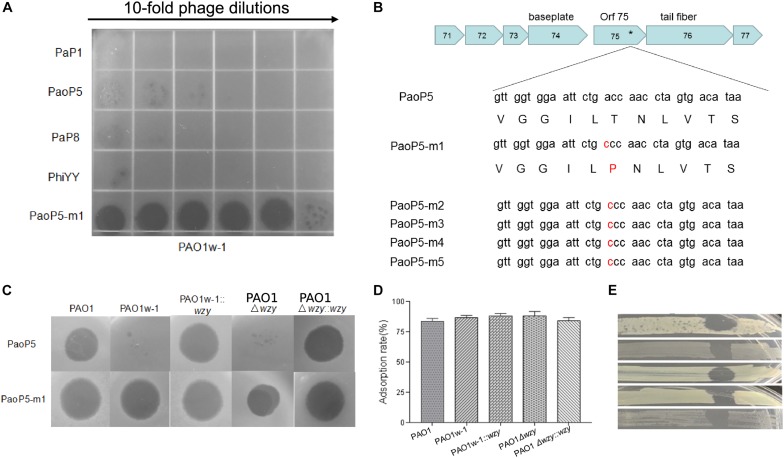
Phage PaoP5-m1 is able to infect *P. aeruginosa* phage-resistant mutants with truncated O-antigen structures. **(A)** Phage spot assay that indicates PaoP5-m1, but not PaP1, PaoP5, PaP8, and phiYY, efficiently infects PAO1w-1. **(B)** PaoP5-m1-5 contain an A715C mutation in *orf75*. PaoP5-m1 efficiently infects **(C)** and adsorbs **(D)** to all the PAO1 derived strains with full or truncated O-antigen structure. **(E)** Phage spot assay indicating five additional *P. aeruginosa* mutants selected by resistance to dsDNA phage PaoP5 are sensitive to PaoP-m1.

PAO1w-1 was highly resistant to the phages PaP1, PaoP5, PaP8, and phiYY (Figure 3A). To attempt to isolate a mutant phage that is able to effectively infect PAO1w-1, 10^7^ plaque forming units (pfu) of PaoP5 was mixed with PAO1w-1, and dozens of mutant phages that overcame resistance and lysed PAO1w-1 were observed. Five mutant phages were selected, purified and sequenced (Table 2). All 5-phage isolates that broke resistance of PAO1w-1 contained a A715C mutation in *orf75*, which was likely to be responsible for the expanded host range (Figure 3B). *orf75* is located between the tail fiber gene and the baseplate gene. Though it is not annotated by blastp, Orf75 was identified as a structural protein by SDS-PAGE and high-performance liquid chromatography – mass spectrometry (Shen et al., 2016). Thus, the product of *orf75* is highly likely to be a receptor-binding protein that is associated with host range.

**TABLE 2:** Primers used in this study.

**Primers**	**Sequence (5′–3′)**
**Validating *wzy* mutation**	
*Wzy*-F1	TCACTGAAACAGGTCGGTAT
*Wzy*-R1	TACCTATAACAACAGCAATCG
**Knockout of *wzy***	
*Wzy*-K*-*F(*Eco*RI)	gGAATTCCTCGCCGTGCACTTGCTCGT
*Wzy*-K*-*R(*Bam*HI)	cgGGATCCGCCAAACACCTCATGTTCCA
**Complementation of *wzy***	
*Wzy* –C-F(*Kpn*I)	ggGGTACCCTTGCCGTCACTTTCTCCGA
*Wzy* –C-R(*Pst*I)	aaCTGCAGGGAGTTGGCGCATATGCATA
**Complementation of *galU***	
*galU* –C-F(*Bam*HI)	cgGGATCCTATGATCAAGAAATGTC TTTTCCCG
*galU* –C-R(*Pst*I)	aaCTGCAGTCAGTGAGCCTTGCC GGTCTTGT
**Mapping mutations in PaoP5-m1**	
062orf-F1 (structural protein-F1)	TTTTGGAAGACCTACCCCTC
062orf-R1 (structural protein-R1)	GCCTTCCTGAACAACACTGA
063orf-F1	GACATGGCAATGGCGATTAC
063orf-R1	GTCGTAGTTGTAAAGAGCAG
064orf-067orf-F1	CGTCTGTGGTTCCGCATGGC
064orf-067orf-R1	AATTTAAGTTCCCCAGTAAG
068orf-F1 (tape measure protein-F1)	CGGTTTTTTCTCTAATCGGT
068orf-R1 (tape measure protein-R1)	CAAATCTAGACGTTGTGATAACA
069orf-073orf-F1	TAGCGACAGACTTCTAGCTC
069orf-073orf-R1	CTGGTAGGGCGAGCCGGATA
074orf-F1	TGGTAGACATTGTTGCTCAA
074orf-R1	TTCCAGCGAATCCAAAATAG
075orf-F1	ATAGAGATCAAGATCAGTGTAACA
075orf-R1	TCGCTACCTGTATGGGTACG
076orf-F1 (tailfiber1-F1)	TCGAAGCTTATAAATTTGTATTCG
076orf-R1 (tailfiber1-R1)	TCGATAGCAATCTCTACTTGACG
077orf-F1	AAACTGACGTAATGACTGATGC
077orf-R1	GGAATCTTGTCGGAATGGGT
078orf-F1 (tailfiber2-F1)	TAGAGGGCTGTCCCACATCT
078orf-R1 (tailfiber2-R1)	CAGTCTCTGTCCATGTTGACTG
079orf-F1 (endolysin-F1)	GCCAATCGGATATTTCCAGA
079orf-R1 (endolysin-R1)	TTGCGGCAGCTACTACGAG
080-088orf-F1	AGACTGCGCCTTGCAAAG
080-088orf-R1	CAAGCCAGAATACTACGTCTATGA

One of the 5 mutant phages, designated PaoP5-m1, was selected for further study. Interestingly, unlike its parent strain, PaoP5, which did not infect PAO1w-1 or a single-gene inactivation of *wzy* (PAO1Δ*wzy*), PaoP5-m1 was able to adsorb and infect all of the PAO1-derived strains, regardless of the presence of full-length or truncated O-antigen (Figures 3C,D). To test the host range of PaoP5-m1 against a larger panel of PaoP5-resistant mutants, we selected 30 white colonies surviving PaoP5 infection across 3 biological replicates. Strikingly, PaoP5-m1 infected all white mutants collected (Figure 3E), indicating that PaoP5-m1 is an excellent phage to eliminate *P. aeruginosa* mutants with truncated O-antigen structures.

Interestingly, similar phage training was unable to generate a mutant phage that infected PAO1r-1, bearing a complete lack of O-antigen (data not shown). This is likely due to the fact that the structures of the O-antigen and core oligosaccharides are quite different, compared to the structural differences between the full-length and truncated O-antigen (Lam et al., 2011). Thus, although phage mutants can adapt rapidly to a truncated receptor structure, they cannot adapt to a completely novel receptor structure in such a short period of time (Schwartz and Lindell, 2017).

### Generation of a Five-Phage Cocktail With Broad Host Range

**FIGURE 4:**
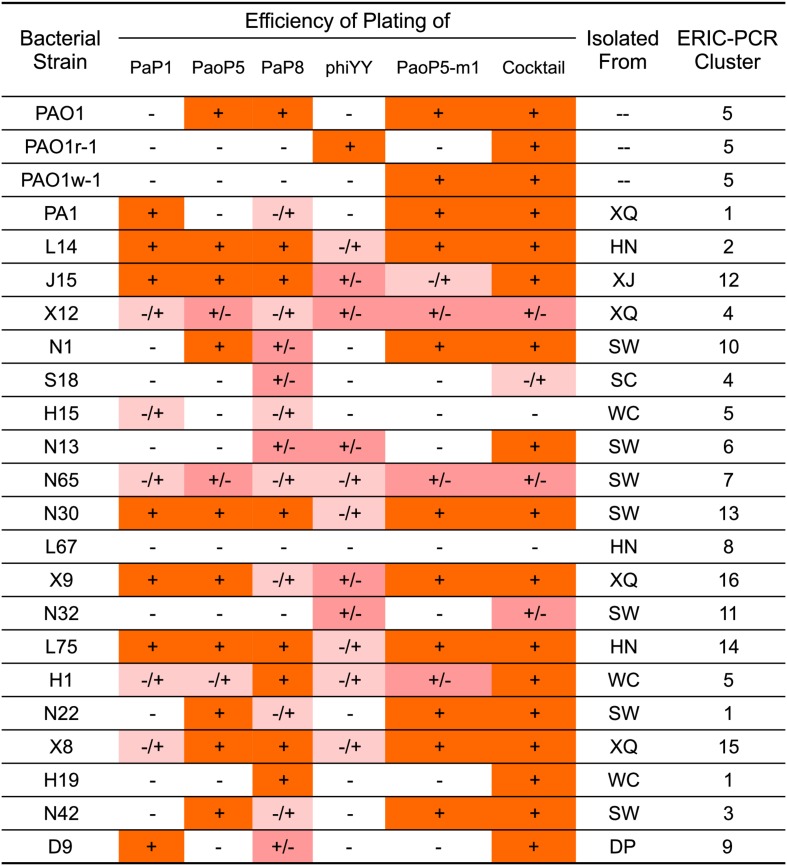
EOP of single phages or a 5-phage cocktail on a panel of 23 *P. aeruginosa* strains. The killing efficiency of each phage strain and cocktail were determined by an EOP assay. (+) = EOP 1; (±) = EOP 0.1–0.01; (–/+) = EOP 0.001; (–) = EOP < 0.0001. For clinical isolates of *P. aeruginosa*, the “Isolated From” column lists the hospital where the strain was isolated: SW, SouthWest Hospital; DP, DaPing Hospital; XQ, XinQiao Hospital; WC, West China Hospital; SC, SiChuan Provincial People’s Hospital; XJ, XiJing Hospital; HN, HeNan Provincial People’s Hospital.

As the genotypes and phenotypes exemplified by PAO1r-1 and PAO1w-1 represented the two major types of resistant mutants which emerged following dsDNA phage treatment of *P. aeruginosa*, phiYY and PaoP5-m1 were included in a 5-phage cocktail we formulated to constrain the emergence of resistance during phage therapy of *P. aeruginosa.* In addition to phiYY and PaoP5-m1, the parent phage of PaoP5-m1, PaoP5, and 2 additional dsDNA phages with a wide host range (PaP1 and PaP8) were included in the phage cocktail. The effectiveness of this cocktail at killing *P. aeruginosa* was examined against a panel of strains that included the type strains PAO1 and PA1, the phage-resistant mutants PAO1-r-1 and PAO1-w-1, as well as 19 *P. aeruginosa* clinical isolates from seven Chinese hospitals (Yang et al., 2016). To maximize the diversity of the panel, the 19 clinical isolates were selected based on diverse Eric-PCR typing (Khosravi et al., 2016; Yang et al., 2016): two strains were selected from clusters 1, 4, and 5, while one strain was selected from each of the remaining 13 typing clusters (Figure 4).

The efficiency of plating (EOP) of each phage and the 5-phage cocktail was measured across the panel of 23 *P. aeruginosa* strains (Figure 4). EOP is more accurate than a simple dot plaque assay in determining phage sensitivity and host range, as the phage lysate is serially diluted to minimize the impact of other antimicrobial agents, such as lysin, within the lysate (Forti et al., 2018). Interestingly, phiYY was able to kill 12 of the strains on the panel, however the EOP was low. Each phage had a distinct host range, and no individual phage lysed all strains in the panel. As expected, the 5-phage cocktail had by far the widest host range and high EOP values.

### The Phage Cocktail Inhibits Bacterial Growth in Liquid Cultures and in Biofilms

**FIGURE 5:**
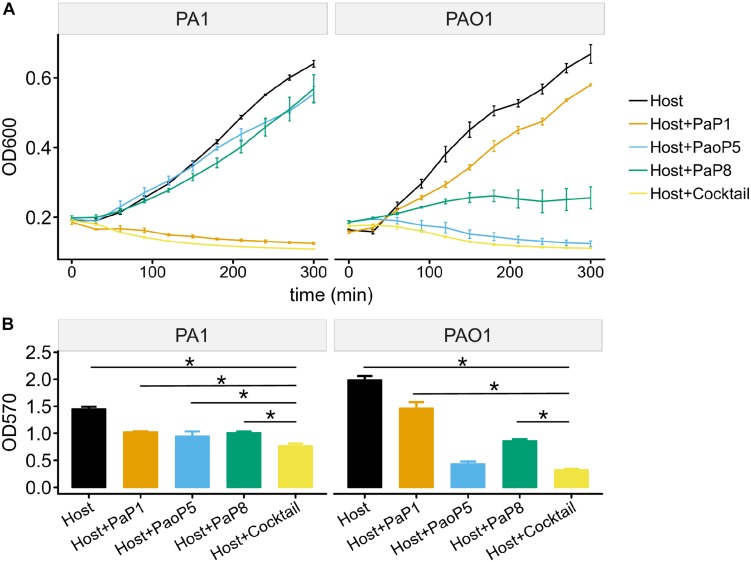
Effects of the 5-phage cocktail on liquid cultures and biofilms of *P. aeruginosa.* **(A)** Growth kinetics of *P. aeruginosa* cells in liquid culture in presence or absence of single phage strains or the phage cocktail. **(B)** Disruption of *P. aeruginosa* biofilms by single phage strains or the phage cocktail. (*P* < 0.05, one-way ANOVA, *n* = 3). The asterisks mark *P*-value of <0.05 as calculated by Student’s *t*-test between two groups.

To test the effects of the individual phages or the phage cocktail on a liquid culture of *P. aeruginosa*, and provide more detail regarding the lysis kinetics of each treatment, cultures of PAO1 or PA1 were infected with each phage or the cocktail, and the optical density (OD_600_) was monitored over 5 h (Figure 5A). For PA1, which is sensitive to PaP1, the addition of PaP1 resulted in a decrease in the OD_600_ of PA1 within 1 h after infection. Meanwhile, challenge with PaP8 did not significantly affect OD_600_ compared to the no phage negative control, which is likely due to the low infection efficiency of PaP8 against PA1, as seen above in Figure 4. In PAO1, phage PaoP5 infection caused a decrease in OD_600_ 1–2 h post-infection, and PaP8 could kill and inhibit PAO1 growth, as indicated by the relatively stable OD_600_. When treated with the 5-phage cocktail, the OD_600_ of both the PAO1 and PA1 cultures decreased about 1 h after phage infection, indicating that the phage cocktail was effective in lysing liquid cultures of both *P. aeruginosa* strains, and it did so in a similar timeframe as the challenge with the single, effective phages.

The capability of phages to reduce biofilms formed by *P. aeruginosa* PA1 or PAO1 was examined using crystal violet staining. Following 24 h of biofilm formation by *P. aeruginosa* strains on 96-well plates, phages were applied individually or in the 5-phage cocktail. Plates were incubated for 4 h, at which point the biofilms were stained with crystal violet. Challenge with the phage cocktail caused a significant reduction in the biofilm biomass of both PA1 and PAO1 (Figure 5B), and eliminated biofilms with a slightly higher efficacy compared to that of challenge using the single, effective phage.

### The Phage Cocktail Inhibits the Development of Phage-Resistant Bacteria in the Short-Term

**FIGURE 6:**
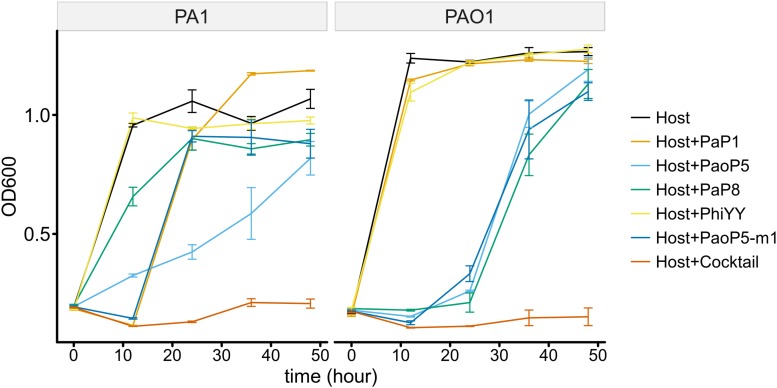
The 5-phage cocktail constrains phage-resistant mutants in the short-term. Growth kinetics of *P. aeruginosa* PA1 and PAO1 in liquid cultures in the presence or absence of single phage strains or the phage cocktail for 48 h.

To determine whether the 5-phage cocktail inhibits the emergence of phage-resistant mutants, *P. aeruginosa* PAO1 or PA1 was mixed with a single phage or the phage cocktail. When treated with a single phage, the OD_600_ of PAO1 and PA1 suffered an initial decrease during the first few hours. However, both strains re-grew to high densities after 12–24 h of incubation, most likely due to the growth of phage-resistant mutants. On the other hand, when treated with the 5-phage cocktail, the OD_600_ of both strains remained low even after 48 h of incubation (Figure 6). This data strongly indicates that the 5-phage cocktail not only efficiently kills *P. aeruginosa*, but also prevents the emergence of phage-resistant mutants *in vitro*.

### After Extended Exposure to the Phage Cocktail, Resistant Isolates Emerge With Mutations in *migA* and *wzy*

**FIGURE 7:**
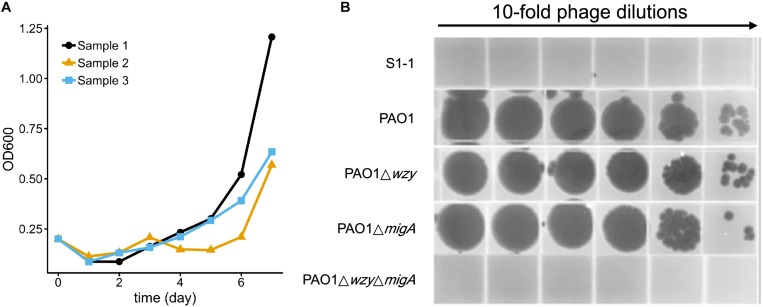
Resistance to the 5-phage cocktail emerges after 7 days, and requires mutations in *wzy* and *migA.* **(A)** Growth kinetics of *P. aeruginosa* PAO1 in liquid culture in the presence of the phage cocktail for 7 days. Three biological repeats were performed. **(B)** Phage spot assay illustrating that the phage cocktail resistant mutant S1-1 and PAO1Δ*wzy*Δ*migA* double mutant are resistant to infection by PaoP5-m1, while single-gene disruptions of PAO1Δ*wzy* and PAO1Δ*migA* are sensitive to PaoP5-m1.

To test whether resistance to the 5-phage cocktail emerged after a longer period of time, the *P. aeruginosa* PAO1 culture containing the phage cocktail was incubated for an additional 7 days without the addition of fresh growth media (Figure 7A). In all three replicates, bacterial density finally began to increase after around 5 days (OD_600_ = 0.5∼1.1), indicating that resistance to the phage cocktail had developed. These cultures were plated on LB agar and two colonies were isolated from each plate (Table 3). The EOP of each selected mutant was examined, and all six isolates were indeed resistant to all phages in the cocktail as no plaques were formed. To determine a genetic cause of the phage resistance, these six isolates, and wild type PAO1, were subjected to whole genome sequencing. The sequencing data revealed that all 6 isolates contained mutations in both *wzy* and *migA* (Table 3).

**TABLE 3:** Mutations found in phage cocktail resistant mutants.

**Sample**	**Nucleotide change**	**Amino acid change**	**Affected gene**	**Affected protein**	**Function**
S1-1/S1-2	T→G	H123P	*migA*	alpha-1,6-rhamnosyltransferase	Uncapped core
	(T)_7→9_	Shift (130/1317 nt)	*wzy*	B-band O-antigen polymerase	OSA
S2-1	G→A	Q42*	*migA*	alpha-1,6-rhamnosyltransferase	Uncapped core
	(CTGGCC)_2→3_	coding (1353/1689 nt)	*fimL*	hypothetical protein	Unkonwn
	G→A	P73S	*wzy*	B-band O-antigen polymerase	OSA
	(CCA)_2→3_	coding (95/972 nt)	gmd	GDP-mannose 4,6-dehydratase	CPA
S2-2	G→A	Q42*	migA	Alpha-1,6-rhamnosyltransferase	Uncapped core
	(CTGGCC)_2→3_	coding (1353/1689 nt)	fimL	hypothetical protein	Unkonwn
	G→A	P73S	wzy	B-band O-antigen polymerase	OSA
	C→A	G31C	gmd	GDP-mannose 4,6-dehydratase	CPA
S3-1	C→T	R217Q	migA	Alpha-1,6-rhamnosyltransferase	Uncapped core
	G→A	P73S	wzy	B-band O-antigen polymerase	OSA
	C→T	W151*	rmd	oxidoreductase Rmd	CPA
S3-2	C→T	R217Q	migA	Alpha-1,6-rhamnosyltransferase	Uncapped core
	G→A	P73S	wzy	B-band O-antigen polymerase	OSA
	G→A	W100*	PA5455	hypothetical protein	CPA

PAO1 was infected with the phage cocktail in three biological repeats. Two phage cocktail resistant mutants S1-1 and S1-2 selected from sample 1 share the same mutation sites in *migA* and *wzy*. The other four mutants have additional mutations in *gmd, rmd* or *PA5455*, which are involved in CPA synthesis. * indicates nonsense mutation.

Many *P. aeruginosa* simultaneously synthesize multiple forms of lipopolysaccharide (Figure 2). These include the capped core glycoforms O-antigen-specific (OSA) and common polysaccharide antigen (CPA), as well as the uncapped core. The enzyme encoded by *migA* is responsible for the transfer of an L-rhamnose to glucose II of the core polysaccharide, and is required for the synthesis of uncapped core. Thus, the *migA* mutant lacks the L-rhamnose residue on the uncapped cores (Kocincova et al., 2012), and the *wzy* mutant lacks the OSA and produces “core + 1” LPS (Li et al., 2018). The sequencing data presented here implies that mutations in both *wzy* and *migA* are required for phage cocktail resistance. As expected, although both PAO1Δ*wzy* and PAO1Δ*migA* were sensitive to PaoP5-m1, a PAO1Δ*wzy*Δ*migA* double mutant was resistant to PaoP5-m1 (Figure 7B). This data implies that PaoP5-m1 may use either core antigen or OSA as receptors.

Among the six selected isolates resistant to the 5-phage cocktail, four had additional mutations. These mutations were located in the genes *gmd, rmd* or *PA5455* (Table 3), all of three of which are involved in CPA synthesis (Lam et al., 2011; Hao et al., 2013). These mutations are less likely to be involved in the resistance to the phage cocktail seen in these strains, as the PAO1Δ*wzy*Δ*migA* double mutation was sufficient to confer resistance to the phage cocktail.

## Discussion

*Pseudomonas aeruginosa* is an opportunistic pathogen which poses a serious health threat, particularly to patients with cystic fibrosis or traumatic burns, as well as the immunocompromised. *P. aeruginosa* is a master of antibiotic resistance, with intrinsic resistance to several drugs via low membrane permeability and expression of efflux pumps, and has a remarkable capacity to mutate and horizontally acquire additional traits leading to multidrug-resistant (MDR) and extensively-resistant (XDR) strains (Lopez-Causape et al., 2018). With the emergence of these phenotypes, which severely limit current treatment options, phage therapy is once again being explored as a potential therapeutic. However, phage therapy is still not widely used due to legislation issues and the lack of proper clinical trials and efficacy studies. As to the phage agents, one of the major barriers to successful phage therapy is the rapid emergence of phage-resistant mutants (Rohde et al., 2018).

Co-evolution of phages and their hosts is common in the natural environment, and the mutation and modification of receptors and receptor binding proteins by both phage and their hosts is well-understood as a major mechanism for this classic Red Queen phenomenon (Paterson et al., 2010). Indeed, one of the major mechanisms of phage resistance in *P. aeruginosa* is modifications to the LPS molecules exposed on the cell surface. As mutant phages with an expanded or altered host range can be isolated through phage training, and serve as complementary components of a therapeutic phage cocktail to prevent emergence of phage-resistant mutants, the goal of this study was to further characterize phage-resistant mutants of *P. aeruginosa*, perform phage training to isolate phages that break these resistant phenotypes, and develop a phage cocktail effective at constraining the development of phage-resistant *P. aeruginosa* during treatment.

The mutant strain PAO1r-1, derived from *P. aeruginosa* PAO1, was resistant to dsDNA phages, but is susceptible to infection by the dsRNA phage, phiYY (Figure 1). This is due to the fact that PAO1r-1 contains a large deletion, including *galU*, which leads to truncated LPS, with core oligosaccharides exposed. phiYY appears to utilize these core oligosaccharides as the receptor for infection (Figure 1). Further credence for this hypothesis was provided by the fact that phiYY did not infect or adsorb the parent strain with a complete LPS, or a the complement strains PAO1r-1::*galU* or PAO1Δ*galU::galU* (Figure 1).

dsRNA phages are a unique group of phages, which are classified in the Cystoviridae family (Mantynen et al., 2018). Currently, there are only 7 sequenced dsRNA phages, and phiYY is the only one that infects human pathogen (Yang et al., 2016), while other 6 dsRNA phages all infects *Pseudomonas syringae*. phi6 is the first isolated dsRNA phage, and serves as an excellent model to study the biology of dsRNA phage (Bamford et al., 1987), while our study demonstrated the antimicrobial potential of dsRNA phage phiYY, which eliminates the brown mutants after infection with dsDNA phages. Moreover, brown mutants are also frequently detected from cystic fibrosis patients with chronic *P. aeruginosa* infections, suggesting the brown mutants can better survive in chronic infection (Smith et al., 2006; Hocquet et al., 2016). Thus, dsRNA phage phiYY might be an efficient agent to kill the brown mutants in chronic *P. aeruginosa* infections, which needs further investigation.

Meanwhile, mutations in another locus involved in LPS production, *wzy*, generated phage-resistant mutants in *P. aeruginosa* PA1, which express a truncated “core + 1” LPS with only one repeat of the O-antigen trisaccharide moiety. Similar phage-resistant *wzy* mutants were obtained in this study from a PAO1 genetic background (Figure 3). Phage training of PaoP5 generated mutant phages capable of breaking the resistance afforded to PAO1 by the *wzy* mutation harbored by PAO1w-1, including the phage isolate, PaoP5-m1 (Figure 3). PaoP5-m1 contains a mutation in *orf75*, and was capable of adsorbing to and infecting PAO1 and all tested *wzy* mutants that arose following infection of PAO1 with PaoP5 (Figure 3). Phage-host co-evolution could expand the host range of the phages and increase the resistance of evolved bacteria (Paterson et al., 2010). Thus, mutant phages with expanded or altered host range can be isolated through phage training, and serve as auxiliary components of a phage cocktail to eliminate the phage resistant bacteria. However, a similar phage training approach failed to produce phage that could break the resistance phenotype of PAO1r-1, likely because the exposed LPS core oligosaccharides of PAO1r-1 are too structurally disparate from the full-length LPS.

A 5-phage cocktail was generated containing three *P. aeruginosa* phages with a broad host range (PaP1, PaoP5, PaP8), as well as the two phages capable of breaking *P. aeruginosa* phage resistance mechanism described above (phiYY and PaoP5-m1). This phage cocktail was examined against a panel of *P. aeruginosa* strains including two laboratory type strains relevant to this study (PAO1 and PA1), the phage-resistant mutants described above (PAO1r-1 and PAO1w-1), and 19 clinical isolates which were selected to represent the 15 Eric-PCR typing clusters. The phage cocktail was able to infect and kill ∼90% of the strains tested, higher than each of the components of the cocktail individually, and did so with increased EOP values (Figure 4). The phage cocktail was effective in killing *P. aeruginosa* in liquid cultures and biofilms, in addition to solid media (Figure 5). While the phage cocktail was effective in constraining the emergence of phage-resistant mutants during the time frames utilized to develop the phage-resistant mutants described above, extended incubation out to 5–7 days led to growth of mutants resistant to the cocktail. All isolated mutants which were resistant to the cocktail had mutations in both *wzy* and *migA*. This development of resistance requiring mutations in multiple loci is a classic illustration of a Red Queen dynamic.

In the natural environment, phage-host co-evolution dynamics have been described as arms race fluctuation selection. It is intriguing to see that this study, while the inclusion of phiYY and PaoP5-m1, the two phages that target two main types of phage resistant mutants in *P. aeruginosa*, significantly constrained the emergence of phage resistant mutants, mutants carrying double or triple gene mutations, including *wzy* and *migA* eventually appeared after 5 days’ incubation. Thus, a better understanding of the mechanism of how these mutants evolved even after long term incubation is important in designing better phage-therapy strategy, and warrant further investigation.

## Experimental Procedures

### Bacterial Strains, Phages and Culture Conditions

The bacterial strains and phages in this work are listed in Table 1. *P. aeruginosa* strains were grown on Luria-Bertani (LB) broth at 37°C. When required, gentamicin (50 μg/ml), or tetracycline (35 μg/ml) were used at a final concentrations in LB broth as described below. To completely remove bacteria, dsDNA phages were treated with chloroform, while dsRNA phage was filtered multiple times through 0.22 μm filters (Millipore), because phiYY is chloroform sensitive. To generate the phage cocktail, 5-phages with the same titer were mixed to a final titer of 10^9^pfu/ml.

### Selection of Phage-Resistant Mutants

The process of isolating phage-resistant mutants and calculating the frequency of resistance has been described in detail previously (Shen et al., 2018). PAO1r-1 was previously isolated, tested and sequenced (Shen et al., 2018). To isolate a PAO1-derived white-phenotype mutant PAO1w-1, PAO1 was infected with PaoP5 and immediately plated on LB agar plates for 24 h. Then, the white mutants were isolated. The *wzy* gene was PCR amplified with primers listed in Table 2, and the PCR products were sequenced to identify the mutation site in *wzy*.

### Construction of *P. aeruginosa* Strains

The knockout and complementation of *galU* and *wzy* were performed as previously described (Le et al., 2014; Li et al., 2018). Briefly, to complement *galU* in PAO1r-1 and PAO1Δ*galU*, the *galU* gene was PCR amplified using primers galU–C-F and galU–C-R, and the PCR product was digested by *Bam*HI/*Pst*I, and cloned into the *Bam*HI/*Pst*I digested plasmid pUCP24 to generate pucp-*galU*. Then, pucp-*galU* was electroporated into PAO1r-1 or PAO1Δ*galU*. The sample approach was applied to complement *wzy* in PAO1w-1 and PAO1Δ*wzy* with primers Wzy–C-F and Wzy–C-R.

To make insertional deletion of *wzy* in PAO1 and PAO1Δ*migA*, a fragment within *wzy* was amplified with primers Wzy-K-F and Wzy-K-R, and the PCR product was digested by *Bam*HI/*Eco*RI, and cloned into the *Bam*HI/*Eco*RI digested plasmid pEX18Gm to generate plasmid pEX-*wzy*. Then, pEX-*wzy* was electroporated into PAO1 or PAO1Δ*migA* to generate insertional mutant.

### Bacteriophage Adsorption Assay

A bacteriophage adsorption assay with various *P. aeruginosa* strains was performed according to a previously described protocol (Le et al., 2014). Briefly, the log phase bacterial cultures were harvested and resuspended in LB broth with an OD_600_ of 0.5. Phage was then added at an MOI of 0.01, and the adsorption proceeded at 37°C for 5 min. Then, 1 ml samples were collected and centrifuged at 16,000 × *g* for 1 min. The supernatant was filtrated using 0.22-μm-pore-size filters (Millipore). Then, the phage titer in the supernatant (t1) and the original phage stock (t0) were determined using double-agar plating assays. The phage adsorption rate was calculated as (t0−t1)/t0. The values of adsorption rate shown in the bar graph are the means and standard deviations are the error bars from three biological replicates.

### EOP Assay and Phage Spot Assay

The EOP of each phage isolate and the 5-phage cocktail on *P. aeruginosa* strains was determined according to a previously described protocol (Forti et al., 2018). 5 μl of serial 10-fold dilutions of phage were spotted on double layer agar plates, on which a specific bacterial host was spread in the upper soft agar. The number of plaques observed after overnight incubation were compared to the number obtained on sensitive strain PAO1, PA1 or PAO1r-1. The phage spot assay is similar to the EOP assay. Mix 200 μl of bacterial culture with 4 ml of soft agar and pour in the agar plate. Then drop 1 μl of phage on the soft agar and observe the formation of plaque after overnight incubation.

### Isolation and Sequencing of Mutant PaoP5 Phages

The process of phage training to break resistance has been described previously (Le et al., 2013). Briefly, ∼10^7^ pfu of phage PaoP5 was mixed with 200 μl of log phase *P. aeruginosa* strain PAO1 (OD_600_ = 0.5) and plated in a double-layer plate. Dozens of plaques appeared after overnight culture. Five clear plaques were randomly selected and transferred into the LB medium. After serial 10- fold dilutions, the plaque assay was repeated with 10 μl of each dilution to obtain well-separated plaques. The same procedure was repeated twice to purify each mutant phage. After purification of mutant phage, genomic DNA was extracted as previously described (Le et al., 2013). Subsequently, the whole region encoding the structural proteins (Orf62 to Orf 88) was amplified by PCR using the primers indicated in Table 2. PCR amplicons were then purified and sequenced using Sanger technology. The mutation site was determined by blast the sequencing data with PaoP5 genome (GeneBank accession number KU297675).

### Biofilm Disruption Experiment

Crystal violet staining was used to monitor biofilm disruption (Forti et al., 2018). An overnight culture of *P. aeruginosa* was diluted to 0.02 OD_600_ in LB broth in 96-well polystyrene microtiter plates, which were then incubated at 37°C for 24 h. The broth was removed and the wells were washed with 200 μl of LB. Then, 200 μl of LB or LB containing phage or phage cocktail was added, and incubated for 4 h at 37°C. Then, crystal violet staining was applied as previously described (Forti et al., 2018). Three biological replicates were performed.

### Selection of Mutants Resistant to the Phage Cocktail

100 μl of phage cocktail was added into 5 ml exponential phase PAO1 (OD_600_ = 0.2). The culture was incubated at 37°C for 7 days with shaking, and the OD_600_ was monitored.

### Bacterial Genome Sequencing

Bacterial genomic DNA was extracted from six mutants which were resistant to the phage cocktail and wild type PAO1 using the UNlQ-10 Column Bacterial Genomic DNA Isolation Kit (Sangon Biotec), and then sequenced using an Illumina Hiseq 2500 platform (∼1 Gbp/sample). Fastp (Chen et al., 2018) was used for adapter trimming and quality filtering after de-multiplexing the raw reads. Mutations were identified in clean reads using Breseq (Barrick et al., 2014) with PAO1 (GenBank accession: NC_002516.2) as the reference genome. The sequence data is available in the NCBI Sequence Read Archive under SRA accession number PRJNA517283.

### Statistical Analysis

The statistical analysis was performed using One-way ANOVA or student’s *t* test. A *P* value < 0.05 was considered as statistically significant.

## Data Availability Statement

Publicly available datasets were analyzed in this study. This data can be found here: NCBI Sequence Read Archive under SRA accession number PRJNA517283.

## Author Contributions

SL and FH conceived the study. YY, WS, and QZ performed the experiments. QC, KX, XJ, and JW analyzed the data. SL, XH, and JB wrote the manuscript. All authors read and approved the final manuscript for publication.

## Conflict of Interest

The authors declare that the research was conducted in the absence of any commercial or financial relationships that could be construed as a potential conflict of interest.
